# Protective Effect of* Diospyros kaki* against Glucose-Oxygen-Serum Deprivation-Induced PC12 Cells Injury

**DOI:** 10.1155/2016/3073078

**Published:** 2016-01-28

**Authors:** Fatemeh Forouzanfar, Shaghayegh Torabi, Vahid R. Askari, Elham Asadpour, Hamid R. Sadeghnia

**Affiliations:** ^1^Neurocognitive Research Center, School of Medicine, Mashhad University of Medical Sciences, Mashhad 917794-8564, Iran; ^2^Pharmacological Research Center of Medicinal Plants, School of Medicine, Mashhad University of Medical Sciences, Mashhad 917794-8564, Iran; ^3^Department of Pharmacology, School of Medicine, Mashhad University of Medical Sciences, Mashhad 917794-8564, Iran; ^4^Anesthesiology and Critical Care Research Center, Shiraz University of Medical Sciences, Shiraz, Iran

## Abstract

Ischemic cerebrovascular disease is one of the most common causes of death in the world. Recent interests have been focused on natural antioxidants and anti-inflammatory agents as potentially useful neuroprotective agents.* Diospyros kaki* (persimmon) has been shown to exert anti-inflammatory, antioxidant, and antineoplastic effects. However, its effects on ischemic damage have not been evaluated. Here, we used an* in vitro* model of cerebral ischemia and studied the effects of hydroalcoholic extract of peel (PeHE) and fruit pulp (PuHE) of persimmon on cell viability and markers of oxidative damage mainly intracellular reactive oxygen species (ROS) induced by glucose-oxygen-serum deprivation (GOSD) in PC12 cells. GOSD for 6 h produced significant cell death which was accompanied by increased levels of ROS. Pretreatment with different concentrations of PeHE and PuHE (0–500 *μ*g/mL) for 2 and 24 h markedly restored these changes only at high concentrations. However, no significant differences were seen in the protection against ischemic insult between different extracts and the time of exposure. The experimental results suggest that persimmon protects the PC12 cells from GOSD-induced injury via antioxidant mechanisms. Our findings might raise the possibility of potential therapeutic application of persimmon for managing cerebral ischemic and other neurodegenerative disorders.

## 1. Introduction

Oxidative stress plays an important role in nerve cell damage and is closely related to the pathogenesis of many central nervous system diseases such as cerebral ischemia, Alzheimer's disease, and Parkinson's disease. During neuropathological states, excessive reactive oxygen species (ROS) generation caused significant damage to cellular macromolecules (i.e., cellular lipids, proteins, or DNA), leading to cellular death [1]. Deprivation of neurons from glucose-oxygen-serum deprivation (GOSD) is a reliable* in vitro* model for understanding of the molecular mechanisms of ischemia-induced neuronal damage and also for the discovery and development of novel compounds for better treatment of cerebral ischemia [2].

Recently, there are intensive interests towards neuroprotective properties of herbal products in ischemic brain injury because of relatively high therapeutic value and less serious side effects [3]. Persimmon (*Diospyros kaki*), which belongs to the Ebenaceae family, is a deciduous small tree native to Eastern Asia and has also been cultivated in Northeastern India, Middle East, Spain, and many other regions. The dry residue of persimmon fruit is known to have many bioactive compounds such as flavonoids, polyphenol (especially tannins), carotenoids, dietary fibers, and minerals [4, 5]. In many traditional medicinal systems, the extract of persimmon fruit is used as antitussive, carminative, and sedative agent and to heal bronchial complaints and hypertension [4, 5]. Recently, it has been shown that persimmon possesses several pharmacological activities such as strong radical scavenging and antioxidant properties [6] and antigenotoxic [6] and anticarcinogenic [7, 8] and anti-inflammatory [9] and antihypertensive [10] and antidiabetic [11] effects.

To our knowledge, no study has previously investigated the effect of persimmon fruit against cerebral ischemia,* in vitro* or* in vivo*. Considering that persimmon has antioxidant and anti-inflammatory properties, the aim of the present study was to investigate the effects of hydroalcoholic extracts of persimmon fruit peel and pulp on GOSD-induced PC12 cells injury. Role of intracellular ROS was also investigated.

## 2. Material and Methods

### 2.1. Cell Line and Reagents

A PC12 cell line was obtained from Pasteur Institute (Tehran, Iran). High glucose Dulbecco's Modified Eagles Medium (DMEM, 4.5 g/L) and fetal calf serum (FCS) were purchased from Gibco (Carlsbad, CA). Glucose-free DMEM, 3-(4,5-dimethylthiazol-2-yl)-2,5-diphenyl tetrazolium (MTT), 2′,7′-dichlorodihydrofluorescein diacetate (H_2_DCF-DA), and other cell culture materials were purchased from Sigma (St. Louis, MO).

### 2.2. Preparation of PuHE (Pulp Hydroalcoholic Extract) and PeHE (Peel Hydroalcoholic Extract)

Fresh fruits of a commercial cultivar in Khorasan province (Iran) were harvested and authenticated by herbarium of Ferdowsi University of Mashhad (Mashhad, Iran, voucher specimen number 11-0203-1).

The pulps and peels of whole seedless fruits were separated, washed, and then shaken with ethanol (70%) and water for 2 days. The resulting extracts were then filtered and concentrated under reduced pressure to get pulp hydroalcoholic extract (PuHE) and peel hydroalcoholic extract (PeHE), respectively. The yields were found to be about 27% w/w.

Stock solutions of PuHE and PeHE were prepared in deionized water and desired working concentrations were made from the stock using complete medium.

### 2.3. Cell Culture

PC12 cells were cultured in DMEM supplemented with 10% FCS and 100 units/mL of penicillin/streptomycin. All cells were maintained in a humidified atmosphere containing 5% CO_2_ at 37°C [12].

### 2.4. Induction of Cell Injury by GOSD

To mimic the ischemic condition, PC12 cells were placed into in an incubation chamber containing “37°C, 5% CO_2_, and 95% N_2_” and cultured in glucose- and serum-free DMEM supplemented with 100 U/mL penicillin and 100 U/mL streptomycin for 6 h [2].

### 2.5. Cell Proliferation (MTT) Assay

MTT was used to identify viable cells which reduce it to a violet formazan dye [13]. PC12 cells (5000/well) were seeded out in 96-well tissue culture plates and, after 24 h, the cells were pretreated with PuHE and PeHE (0–500 *μ*g/mL) for 2 and 24 h and then subjected to GOSD insult for 6 h, respectively. The concentrations and times were chosen based on earlier experiments. Controls were treated identically. At 6 h after GOSD insult, MTT was added to each well to achieve a final concentration of 0.5 mg/mL. After incubation at 37°C for 4 h, the medium was removed and 100 *μ*L of dimethyl sulfoxide was added to each well and kept for 10 min and the absorbance at 570 and 620 nm (background) was measured using StatFAX303 plate reader. All experiments were carried out in triplicate; the percentage of viable cells was calculated as the mean ± SEM with controls set to 100%.

### 2.6. Measurement of Intracellular Reactive Oxygen Species (ROS)

The determination of intracellular ROS levels was accomplished with a fluorescent probe, H_2_DCF-DA, as described previously with minor modifications [12]. H_2_DCF-DA readily diffuses through the cell membrane and is enzymatically hydrolyzed by intracellular esterases to nonfluorescent H_2_DCF, which is then rapidly oxidized to highly fluorescent DCF (2′,7′-dichlorofluorescin) in the presence of ROS. The DCF fluorescence intensity is believed to parallel the amount of intracellular ROS [14]. In brief, PC12 cells (10^4^ cells/well) were pretreated with different concentrations of PuHE and PeHE (0–500 *μ*g/mL) for 2 h and then subjected to GOSD for 6 h in which the same treatments were applied. At 6 h after ischemic insult, the cells were incubated with 20 *μ*M H_2_DCF-DA at 37°C for 30 min in the dark. The DCF fluorescence intensity was detected using a FLUO-star galaxy fluorescence plate reader (Perkin Elmer 2030, Multilabel reader, Finland) with excitation wavelength set at 485 nm and emission wavelength set at 530 nm.

### 2.7. Statistical Analysis

The results are presented as the mean ± standard error (SEM). The values were compared using the one-way analysis of variance (ANOVA) followed by Tukey's post hoc test for multiple comparisons. The *p* values less than 0.05 were considered to be statistically significant.

## 3. Results

### 3.1. Effects of PeHE and PuHE on Cell Viability following GOSD Insult in PC12 Cells

Exposure to GOSD for 6 h significantly decreased cell viability as compared with control cells. The average survival rate of cells under the GOSD condition was about 36%.

**Figure 1:**
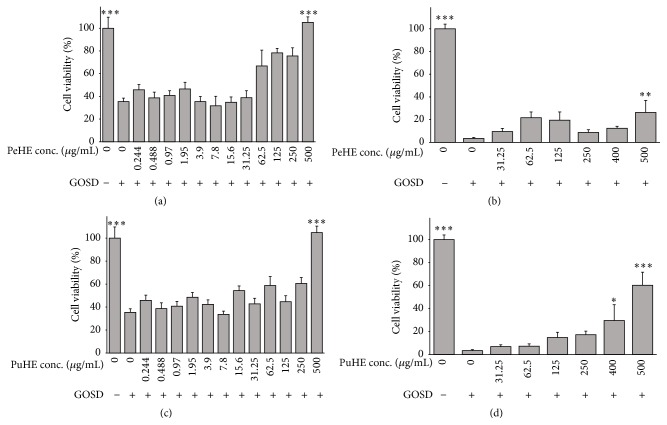
Effects of hydroalcoholic extracts of fruit peel (PeHE) and pulp (PuHE) of* Diospyros kaki* on PC12 cells death induced by glucose-oxygen-serum deprivation (GOSD). PC12 cells were treated with PeHE for 2 h (a) and 24 h (b) or PuHE for 2 h (c) and 24 h (d) and then subjected to GOSD for 6 h. the data are presented as mean ± SD from independent experiments performed in triplicate. ^*∗*^ *p* < 0.05, ^*∗∗*^ *p* < 0.01, and ^*∗∗∗*^ *p* < 0.001 as compared to ischemic group.

Pretreatment with PeHE for 2 h significantly attenuated GOSD-induced damage to PC12 cells only at high concentration (500 *μ*g/mL; 110.5 ± 4.2; *p* < 0.001) as compared to GOSD group (37.0 ± 4.0) (Figure 1(a)). Also, pretreatment with PeHE for 24 h significantly attenuated GOSD-induced PC12 cells death at concentration of 500 *μ*g/mL (27.3 ± 10.0; *p* < 0.01) (Figure 1(b)).

In the same way, pretreatment with PuHE for 2 h significantly attenuated cell death induced by GOSD at concentration of 500 *μ*g/mL (105.0 ± 5.3; *p* < 0.001) as compared to GOSD group (35.0 ± 2.0) (Figure 1(c)). Again, pretreatment with PuHE for 24 h significantly increased cell survival following ischemic insult at concentration of 500 *μ*g/mL (57.5 ± 9.5; *p* < 0.001) and at concentration of 400 *μ*g/mL (29.0 ± 13.0; *p* < 0.05) (Figure 1(d)).

### 3.2. Effects of PeHE and PuHE on ROS Production following GOSD Insult in PC12 Cells

**Figure 2:**
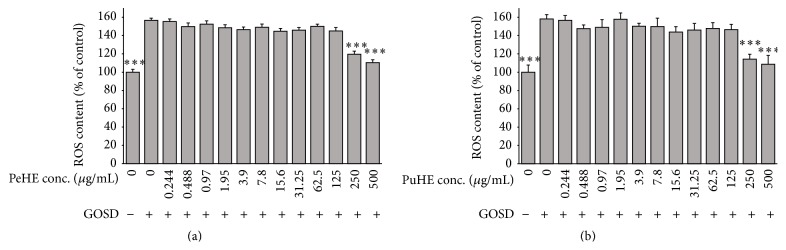
Effects of hydroalcoholic extracts of fruit peel (PeHE) and pulp (PuHE) of* Diospyros kaki* on ROS production in glucose-oxygen-serum deprivation- (GOSD-) treated PC12 cells. PC12 cells were preincubated with PeHE (a) or PuHE (b) for 2 h prior to GOSD insult. Then, PC12 cells were loaded with DCF-DA, and intracellular ROS were detected by fluorimetry. The data are represented as mean ± SD from independent experiments performed in triplicate. ^*∗∗∗*^ *p* < 0.001 as compared to ischemic group.

GOSD significantly increased the number of DCF-positive cells illustrating an elevation of ROS production to 156%. As shown in Figure 2(a), pretreatment with PeHE for 2 h resulted in a significant (*p* < 0.001) reduction in ROS production following GOSD (121.4 ± 2.0 and 110.4 ± 2.0 at concentrations of 250 and 500 *μ*g/mL, resp.).

As illustrated in Figure 2(b), pretreatment with PuHE for 2 h caused a significant reduction of ROS production following GOSD (114.3 ± 4.2; *p* < 0.01 and 108.4 ± 9; *p* < 0.001 at concentrations of 250 and 500 *μ*g/mL, resp.).

## 4. Discussion

According to our knowledge, this is the first report of the neuroprotective effect of* D. kaki* fruit extract. In the present study, we demonstrated that pretreatment with peel and pulp fruit extracts of* D. kaki* was able to promote cell survival and depress ROS increase upon GOSD stress in PC12 cells. Recently, substantial efforts have been invested for identifying optimal drugs that can reduce ischemic brain damage [2].

It is well known that ischemia increases the formation of ROS in brain tissues and antioxidants and ROS scavengers can decrease tissue damage following ischemic injury [15–17]. Excessive production of ROS will act as damaging molecule causing cell death either directly, through interacting and destroying cellular proteins, lipids, and DNA, or indirectly, by affecting normal cellular signaling pathways and gene regulation [15, 18].

One possible mechanism underlying the attenuation of ROS generation following ischemic insult is due to antioxidant and free radical scavenger properties of persimmon. Previous studies have shown that flavonoids from persimmon peel and leaf could protect against hydrogen proxide-induced oxidative damage,* in vitro* [19, 20]. High molecular weight persimmon tannin also ameliorates oxidative damage and cognition deficits in D-galactose-induced senescent mice [21]. Tian et al. showed that administration of high molecular weight persimmon condensed tannin (HMWPT) significantly protected serum and liver antioxidant enzymes from damage and prevented lipid peroxidation in bromobenzene-treated mice [22]. In addition, several studies suggest that persimmon leaves extract and its flavonoids exhibit neuroprotective effects and protect rats from both focal and global cerebral ischemic injury,* in vivo,* as well as cortical neurons from hypoxia-induced injury,* in vitro* [23, 24]. High-performance liquid chromatography (HPLC) analysis also revealed that gallic acid and quercetin were the major phenolic components in the fruit extracts of persimmon [25].

Also, antiaging and memory enhancing effects of oligomeric proanthocyanidins isolated from persimmon fruits have also been described in a senescence-accelerated mouse prone model [26–28]. A recent study showed that ethanol extract of* Diospyros kaki* leaves significantly attenuated the upregulation of vascular endothelial growth factor, fibroblast growth factor, interleukin-6, and matrix metalloproteinase-2 (MMP-2) protein levels, in alkali burn-induced corneal neovascularization in rats [29]. Kim et al. also showed that ethanol extract of* Diospyros kaki* leaves prevented degenerative retinal diseases in N-methyl-N-nitrosourea- (MNU-) induced retinal degeneration in mice [30].

## 5. Conclusions

In summary, the results of the current study suggest that persimmon is able to protect cultured PC12 cells against damage induced by GOSD. These effects of persimmon are, at least in part, attributable to its antioxidant property. This study on the neuroprotective effects of persimmon may suggest the possible application of persimmon in clinical setting to prevent and treat the common neurological insults.

## References

[1] Shukla V., Mishra S. K., Pant H. C. (2011). Oxidative stress in neurodegeneration. *Advances in Pharmacological Sciences*.

[2] Wang C.-H., Lee W.-J., Ghanta V. K., Wang W.-T., Cheng S.-Y., Hsueh C.-M. (2009). Molecules involve in the self-protection of neurons against glucose-oxygen-serum deprivation (GOSD)-induced cell damage. *Brain Research Bulletin*.

[3] Suk K. (2005). Regulation of neuroinflammation by herbal medicine and its implications for neurodegenerative diseases: a focus on traditional medicines and flavonoids. *NeuroSignals*.

[4] Mallavadhani U. V., Panda A. K., Rao Y. R. (1998). Pharmacology and chemotaxonomy of Diospyros. *Phytochemistry*.

[5] Singh S., Joshi H. (2011). Diospyros kaki (Ebenaceae): a review. *Asian Journal of Research in Pharmaceutical Sciences*.

[6] Jang I.-C., Jo E.-K., Bae M.-S. (2010). Antioxidant and antigenotoxic activities of different parts of persimmon (*Diospyros kaki* cv. Fuyu) fruit. *Journal of Medicinal Plants Research*.

[7] Kawase M., Motohashi N., Satoh K. (2003). Biological activity of persimmon (*Diospyros kaki*) peel extracts. *Phytotherapy Research*.

[8] Achiwa Y., Hibasami H., Katsuzaki H., Imai K., Komiya T. (1997). Inhibitory effects of persimmon (*Diospyros kaki*) extract and related polyphenol compounds on growth of human lymphoid leukemia cells. *Bioscience, Biotechnology and Biochemistry*.

[9] Del Carmen Recio M., Giner R. M., Manez S. (1995). Investigations on the steroidal anti-inflammatory activity of triterpenoids from *Diospyros leucomelas*. *Planta Medica*.

[10] Kawakami K., Aketa S., Sakai H., Watanabe Y., Nishida H., Hirayama M. (2011). Antihypertensive and vasorelaxant effects of water-soluble proanthocyanidins from persimmon leaf tea in spontaneously hypertensive rats. *Bioscience, Biotechnology and Biochemistry*.

[11] Li C., Bei W., Li Y., Lou J. (2007). Ethylacetate extract of *Diospyros kaki* leaf for preventing and treating hyperglycemic, diabetes and metabolic syndromes. *Faming Zhuanli Shenqing Gongkai Shuomingshu*.

[12] Forouzanfar F., Afkhami Goli A., Asadpour E., Ghorbani A., Sadeghnia H. R. (2013). Protective effect of *Punica granatum* L. against serum/glucose deprivation-induced PC12 cells injury. *Evidence-Based Complementary and Alternative Medicine*.

[13] Mosmann T. (1983). Rapid colorimetric assay for cellular growth and survival: application to proliferation and cytotoxicity assays. *Journal of Immunological Methods*.

[14] Wang H., Joseph J. A. (1999). Quantifying cellular oxidative stress by dichlorofluorescein assay using microplate reader. *Free Radical Biology and Medicine*.

[15] Li Y., Bao Y., Jiang B. (2008). Catalpol protects primary cultured astrocytes from in vitro ischemia-induced damage. *International Journal of Developmental Neuroscience*.

[16] Yamada J., Yoshimura S., Yamakawa H. (2003). Cell permeable ROS scavengers, Tiron and Tempol, rescue PC12 cell death caused by pyrogallol or hypoxia/reoxygenation. *Neuroscience Research*.

[17] Tagami M., Yamagata K., Ikeda K. (1998). Vitamin E prevents apoptosis in cortical neurons during hypoxia and oxygen reperfusion. *Laboratory Investigation*.

[18] Chan P. H. (2001). Reactive oxygen radicals in signaling and damage in the ischemic brain. *Journal of Cerebral Blood Flow & Metabolism*.

[19] Lee Y. A., Cho E. J., Yokozawa T. (2008). Protective effect of persimmon (*Diospyros kaki*) peel proanthocyanidin against oxidative damage under H_2_O_2_-induced cellular senescence. *Biological and Pharmaceutical Bulletin*.

[20] Bei W., Peng W., Ma Y., Xu A. (2005). Flavonoids from the leaves of *Diospyros kaki* reduce hydrogen peroxide-induced injury of NG108-15 cells. *Life Sciences*.

[21] Tian Y., Zou B., Yang L. (2011). High molecular weight persimmon tannin ameliorates cognition deficits and attenuates oxidative damage in senescent mice induced by D-galactose. *Food and Chemical Toxicology*.

[22] Tian Y., Zou B., Li C.-M., Yang J., Xu S.-F., Hagerman A. E. (2012). High molecular weight persimmon tannin is a potent antioxidant both ex vivo and in vivo. *Food Research International*.

[23] Bei W., Peng W., Zang L., Xie Z., Hu D., Xu A. (2007). Neuroprotective effects of a standardized extract of *Diospyros kaki* leaves on MCAO transient focal cerebral ischemic rats and cultured neurons injured by glutamate or hypoxia. *Planta Medica*.

[24] Bei W., Zang L., Guo J. (2009). Neuroprotective effects of a standardized flavonoid extract from *Diospyros kaki* leaves. *Journal of Ethnopharmacology*.

[25] Pu F., Ren X.-L., Zhang X.-P. (2013). Phenolic compounds and antioxidant activity in fruits of six *Diospyros kaki* genotypes. *European Food Research and Technology*.

[26] Lee Y. A., Cho E. J., Yokozawa T. (2010). Oligomeric proanthocyanidins improve memory and enhance phosphorylation of vascular endothelial growth factor receptor-2 in senescence-accelerated mouse prone/8. *British Journal of Nutrition*.

[27] Yokozawa T., Lee Y. A., Cho E. J., Matsumoto K., Park C. H., Shibahara N. (2011). Anti-aging effects of oligomeric proanthocyanidins isolated from persimmon fruits. *Drug Discoveries & Therapeutics*.

[28] Yokozawa T., Lee Y. A., Zhao Q., Matsumoto K., Cho E. J. (2009). Persimmon oligomeric proanthocyanidins extend life span of senescence-accelerated mice. *Journal of Medicinal Food*.

[29] Yang S. J., Jo H., Kim K.-A., Ahn H. R., Kang S. W., Jung S. H. (2016). Diospyros kaki extract inhibits alkali burn-induced corneal neovascularization. *Journal of Medicinal Food*.

[30] Kim K.-A., Kang S. W., Ahn H. R., Song Y., Yang S. J., Jung S. H. (2015). The leaves of persimmon (*Diospyros kaki* Thunb.) ameliorate *N*-methyl-*N*-nitrosourea (MNU)-induced retinal degeneration in mice. *Journal of Agricultural and Food Chemistry*.

